# Pharmacological Treatment with Annexin A1 Reduces Atherosclerotic Plaque Burden in LDLR^-/-^ Mice on Western Type Diet

**DOI:** 10.1371/journal.pone.0130484

**Published:** 2015-06-19

**Authors:** Dennis H. M. Kusters, Martijn L. Chatrou, Brecht A. G. Willems, Marijke De Saint-Hubert, Matthias Bauwens, Emiel van der Vorst, Stefania Bena, Erik A. L. Biessen, Mauro Perretti, Leon J. Schurgers, Chris P. M. Reutelingsperger

**Affiliations:** 1 Department of Biochemistry, Cardiovascular Research Institute Maastricht, Maastricht, the Netherlands; 2 VitaK BV, Maastricht University, Maastricht, the Netherlands; 3 Department of Nuclear Medicine, Maastricht University Medical Centre, Maastricht, the Netherlands; 4 Department of Pathology, Cardiovascular Research Institute Maastricht, Maastricht, the Netherlands; 5 William Harvey Research Institute, Barts and The London School of Medicine and Dentistry, Queen Mary University of London, London, United Kingdom; Medical Faculty, Ludwig Maximilians University Munich, GERMANY

## Abstract

**Objective:**

To investigate therapeutic effects of annexin A1 (anxA1) on atherogenesis in LDLR^-/-^ mice.

**Methods:**

Human recombinant annexin A1 (hr-anxA1) was produced by a prokaryotic expression system, purified and analysed on phosphatidylserine (PS) binding and formyl peptide receptor (FPR) activation. Biodistribution of ^99m^Technetium-hr-anxA1 was determined in C57Bl/6J mice. 12 Weeks old LDLR^-/-^ mice were fed a Western Type Diet (WTD) during 6 weeks (Group I) or 12 weeks (Group P). Mice received hr-anxA1 (1 mg/kg) or vehicle by intraperitoneal injection 3 times per week for a period of 6 weeks starting at start of WTD (Group I) or 6 weeks after start of WTD (Group P). Total aortic plaque burden and phenotype were analyzed using immunohistochemistry.

**Results:**

Hr-anxA1 bound PS in Ca^2+^-dependent manner and activated FPR2/ALX. It inhibited rolling and adherence of neutrophils but not monocytes on activated endothelial cells. Half lives of circulating ^99m^Tc-hr-anxA1 were <10 minutes and approximately 6 hours for intravenously (IV) and intraperitoneally (IP) administered hr-anxA1, respectively. Pharmacological treatment with hr-anxA1 had no significant effect on initiation of plaque formation (-33%; P = 0.21)(Group I) but significantly attenuated progression of existing plaques of aortic arch and subclavian artery (plaque size -50%, P = 0.005; necrotic core size -76% P = 0.015, hr-anxA1 vs vehicle) (Group P).

**Conclusion:**

Hr-anxA1 may offer pharmacological means to treat chronic atherogenesis by reducing FPR-2 dependent neutrophil rolling and adhesion to activated endothelial cells and by reducing total plaque inflammation.

## Introduction

Atherosclerosis is a systemic chronic inflammatory disease affecting the vascular wall of arteries and is the major cause of morbidity and mortality in both developed and developing countries. The disease is characterized by confined manifestations of atherosclerotic lesions in the arterial vessel wall that may develop into unstable plaques causing adverse outcomes over time [1–3]. Unstable plaques are characterized by abundant presence of inflammatory cells, a large necrotic core and a thin fibrous cap [4]. Inflammatory cells are considered key players in initiation of- and progression to an unstable plaque [5,6]. Activated neutrophils act as important effectors and regulators of inflammation through their ability to produce a myriad of effector molecules including cytokines, chemokines and angiogenic factors [7]. Reducing recruitment of neutrophils to the inflamed arterial vessel wall by neutrophil-specific antibodies and gene-deletions of chemokines and their receptors, suppresses arterial lesion development in mouse models of atherosclerosis [8–10].

Annexin A1 (anxA1) is a member of the multigene annexin family [11] with potent anti-inflammatory activity [12]. The polypeptide chain of anxA1 comprises a C-terminal core domain, which is conserved amongst all members of the family and harbors the Ca^2+^/phospholipid binding sites, and an N-terminal tail that varies between annexin members. The N-terminal tail of anxA1 can interact with the receptor for formylated peptides (FPR2/ALXR) [11,13–16]. In absence of calcium and a phospholipid surface, the tail is concealed within the core domain. However, upon membrane binding anxA1 changes conformation and exposes the N-terminus at the surface enabling interaction with FPR-2 [15]. AnxA1-FPR2 interaction results in inhibition of neutrophil recruitment to inflamed sites [17]. Alternatively, proteolysis can cause release of the N-terminal peptide [18], which then can interact with FPR1 and 2 evoking anti-inflammatory activity [19]. Pharmacological treatment of inflammation using anxA1 and its N-terminal peptide has been studied in mouse models of neutrophil-dependent edema [20], cardiac ischemia-reperfusion injury [21,22] and acute peritonitis [23], however, hitherto not in atherosclerosis.

Recently it was shown by gene-knockout strategies that the anxA1-FPR2/ALX axis contributes to atherogenesis in apoE^-/-^ mice by mediating recruitment of inflammatory cells to the atherosclerotic plaque [24]. In this paper we studied pharmacological effects of recombinant human recombinant anxA1 (hr-anxA1) on the initiation of atherosclerosis and the progression of established atherosclerotic plaques in an LDLR^-/-^ mouse model. We demonstrate that hr-anxA1 has no significant effect on initiation of early plaque development, but significantly attenuates progression of existing lesions to unstable plaques.

## Methods

### Design, production and purification of recombinant human (hr)-anxA1

cDNA coding for hr-anxA1 was amplified by polymerase chain reaction (PCR) using primers: 5’-GGTATCGAGGGAAGGGCAATGGTATCAGAATTC-3’ and 5’-GCTCAGCTAATTAAGCTTTAGTTTCCTCCACAAAGAGC-3’. The primers introduced Stu-I and Hind-III restriction sites, required for the ligation into the expression vector pQE30Xa (Qiagen). His-tagged hr-anxA1 was produced according to previously published protocol for anxA5 [25]. In short, *E*. *Coli* (SG13009 pREP4) (Novagen) were fermented in Luria-Bertani broth medium supplemented with Ampicillin (50ug/ml, Roche), Kanamycin (30ug/ml, Gibco) and 0.5% glycerol. At OD_450_ of >6, over-expression of the protein was initiated by addition of 5mM isopropyl β-D-1-thiogalactopyranoside (IPTG, Eurogentec). Proteins were purified by IMAC. Purity and homogeneity were assessed by silver-stained SDS-PAGE, western blotting and MALDI-TOF/TOF analysis.

### Endotoxin determination

Endotoxin was determined with the Endosafe-PTS (Charles-River) according to manufacturer’s protocol. Purified protein preparations containing <1 unit/ml endotoxin were used for experiments.

### Ellipsometry

PS-binding property of purified hr-anxA1 was determined by ellipsometry using a bilayer of 20 mole% dioleoyl-phosphatidylserine / 80 mole% dioleoyl-phosphatidylcholine (20 mole% DOPS/80 mole% DOPC; Avanti Polar Lipids, Alabaster, AL, USA) as described previously [26]

### Cells

Primary bone marrow derived monocytes were isolated as described before [27]. Mice were anesthetized with 5% isoflurane before sacrifice by cervical dislocation. Cells were grown for 8 days in RPMI 1640 containing 10 mM Hepes, 10% heat inactivated fetal bovine serum (FBS, Gibco-BRL), 15% L929-cell conditioned medium, 100U/ml Penicillin (Gibco-BRL) and 100 μg/ml Streptomycin (Gibco-BRL) [28].

The human monocytic cell line THP-1 (ATCC, Manassas, USA), was cultured in RPMI 1640 without color indicator (Gibco-BRL, Invitrogen, USA) supplemented with 2mM glutamine (Gibco-BRL, Invitrogen), 10% heat-inactivated FBS, 100units/ml penicillin and 100μg/ml streptomycin.

Polymorphonuclear leukocytes (PMN) were isolated from blood collected into 3.2% sodium citrate, diluted 1:1 in RPMI 1640 (Sigma-Aldrich) before separation through a double-density gradient as described previously [19].

Confluent human umbilical vein endothelial cells (HUVEC, PromoCell, C12203) were grown to a monolayer in a μ-slide (Ibidi, 80666, Germany) before flow experiments.

### Calcium mobilization and flow-chamber assay

Receptor binding studies were performed on FPR-2 transfected HEK293 cells as described previously [29]. Leukocyte rolling and adhesion flow chamber experiments were performed as described previously [19]. Each flow experiment consisted in total of a 10 second recording at 3.3 frames per second. Cells of all 32 frames were counted: adhering cells were stationary in all frames, while rolling cells were defined as all interacting non-stationary cells.

### In vitro internalization of hr-anxA1

Binding and internalization of fluorescently labeled hr-anxA1 by apoptotic Jurkat cells was visualized with confocal laser scanning microscopy (CLSM) as described previously [30].

### Animals

All animal experiments have been conducted under a protocol approved by the ethics committee for animal experiments of Maastricht University (DEC 2012–068) and comply with ARRIVE (Animal Research: Reporting In Vivo Experiments) guidelines and followed the European Union Directive (2010/63/EU). All animal experiments were conducted as humane as possible, a total of 52 animals were used. For pharmacological treatment with hr-anxA1, 12 weeks old LDLR^-/-^ mice were fed a Western type diet (WTD, 0.15% cholesterol) (AB diets 4021.13) for 6 weeks (group I, n = 12) or 12 weeks (group P, n = 12). Mice received 1 mg/kg hr-anxA1 or PBS (control) 3 times per week via intraperitoneal injection during 6 weeks initiating at the start of WTD (group I) or 6 weeks after start of WTD (group P). Mice were sacrificed under anesthesia of 5% isoflurane by vena cava puncture after subcutaneous injection of 0.1 mg/kg Buprenorphine (Temgesic). Aortic arches including major branch points were dissected, fixed in 1% PFA o/n, paraffin embedded and sectioned at 4 μm thickness. Total plaque area was determined for the entire aortic arch and the brachiocephalic, carotid and subclavian arteries using standard hematoxylin/eosin (H&E) staining on every fifth section, and analyzed with ImageJ Software (v1.45). Progression and stability of plaques was scored on the following parameters: neutrophil and macrophage content, apoptosis and necrosis, cap thickness and calcification status (see S1 and S2 Tables).

### Nuclear imaging, biodistribution and blood clearance

Purified His-tagged hr-anxA1 was radiolabeled with technetium-99m (^99m^Tc(CO)_3_-His-anxA1) using the IsoLink tricarbonyl labeling method (Covidien, Petten, The Netherlands) as described previously for His-anxA5 [31]. Nuclear imaging, blood clearance and biodistribution were performed using 12 weeks old LDLR^-/-^ mice (Charles-River). Biodistributions and blood clearances of IV and IP injected 100 MBq ^99m^Tc(CO)_3_-His-anxA1 were determined. To exclude any dietary effect on the biodistribution or blood clearance, mice were put on WTD 2 weeks prior to the experiment. Total body single photon emission tomography (SPECT) imaging was performed 45 minutes post-injection as previously described [32].

### Flow cytometry of blood and bone marrow cells

Single cell suspensions were prepared and stained with anti-CD3,-CD4,-CD8a,-CD11b,-CD115,-Ly6C,-Ly6G,-B220,-NK1.1, or isotype control IgG. Antibodies conjugated to FITC, PE, APC, Cy7, eFluo450, or PerCP (all BD Biosciences) were used and cells were analyzed using a FACS-Canto II and FACSDiva Software (V6.1.3, BD Biosciences).

### Anti-hr-anxA1 antibody formation

Blood was collected in 3.2% sodium citrate and plasma was collected by centrifugation. A 96-well plate was coated with 5 μg/ml hr-anxA1 in 0.1M carbonate buffer (pH 9.5) o/n and blocked with 3% non-fat dry milk in phosphate buffered saline (PBS). Ten times diluted plasma was added and incubated 1h at 37°C, rabbit-α-human anxA1 antibody was used as positive control. HRP-labeled rat-α-mouse-IgG (Dako, P0161) was added and incubated 1h at 37°C. Chromogenic substrate tetramethyl benzidine (TMB) was measured at 450nm.

### Endogenous thrombin potential (ETP)

ETP was determined in plasma containing 3.3 μM corn trypsin inhibitor (CTI) using 7 pM of tissue factor, 60 μM of phospholipids (20%PS/60%PC/20%PE), 420 μM fluorogenic substrate and 8.2 mM Ca^2+^ [33].

### Immunohistochemistry

Immunohistochemistry was performed using antibodies against MAC-3 (BD Biosciences, clone M3/84, 553322) for all macrophages, iNOS (Abcam, AB3523) for M1-polarized and Ym1 (kind gift from professor Christoph Binder, Vienna, Austria) and CD206 (ITK diagnostics, 141702) for M2-polarized macrophages.

### Statistical analysis

All data is presented as mean ± SEM. Normality was tested using the Shapiro-Wilk normality test with GraphPad Prism 5.0 software. Statistical significance between two groups was tested by unpaired two tailed student’s *t*-test. A value of P<0.05 was considered statistically significant. *P*-values are indicated in the tables and figures by asterisks as follows *P<0.05, **P<0.005, ***P<0.001.

## Results

### Physical and biological characterization of hr-anxA1

**Fig 1:**
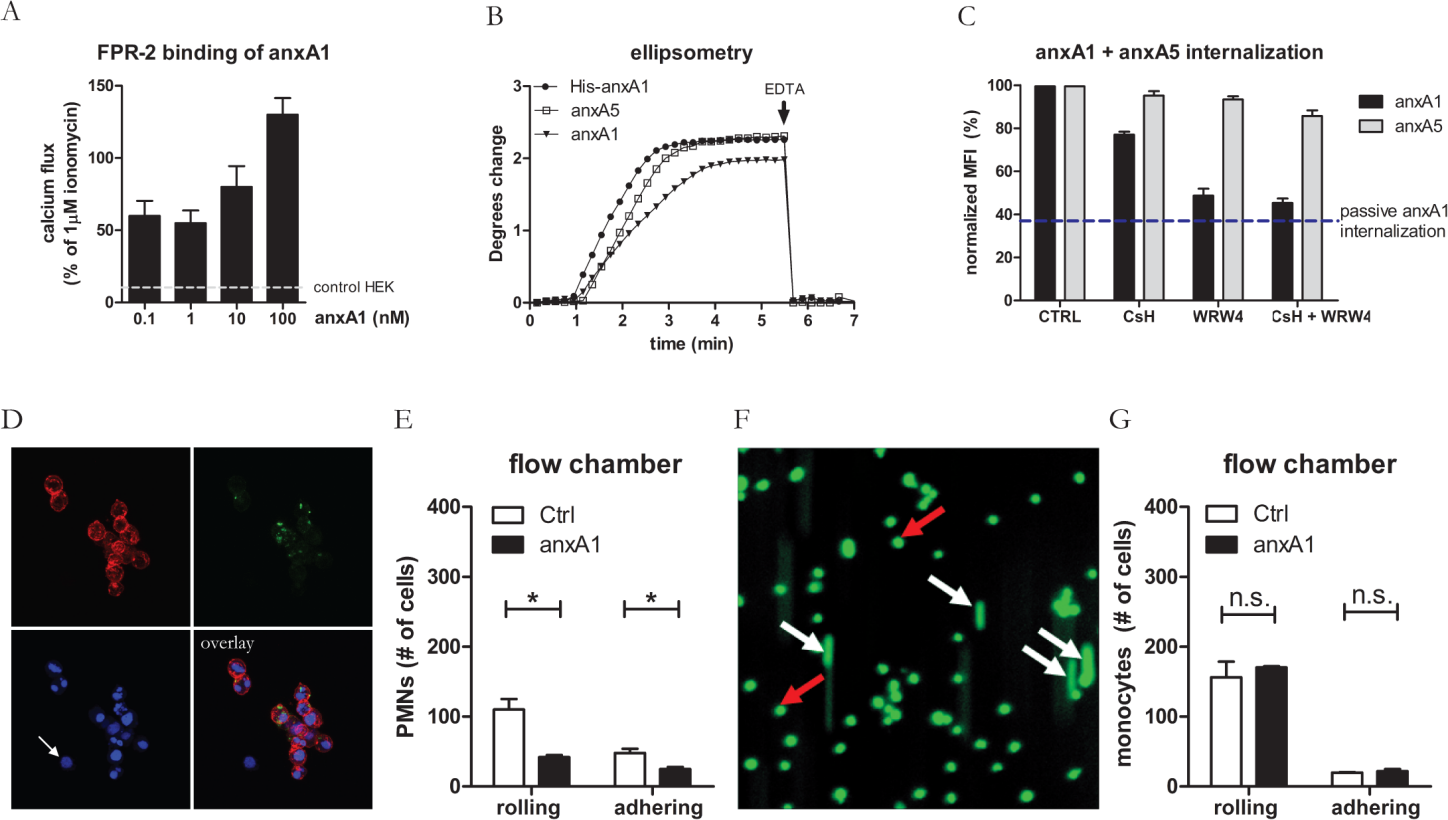
Physical and biological characterization of hr-anxA1. (A) Hr-anxA1 induced concentration dependent calcium flux in FPR2 transfected HEK-293 cells, 1 μM ionomycin is taken as reference value (100%). (B) Ellipsometry analysis shows calcium dependent binding of 1 µg/ml purified annexin to a 20/80 mol% PS/PC bilayer. (C) Internalization of fluorescent annexin by apoptotic Jurkat cells in presence and absence of inhibitors of FPR1 (cyclosporin H, 1 μM) and FPR2/3 (WRW, 10 μM) as analyzed by flow cytometry. Mean fluorescence intensity (MFI) is normalized to MFI of annexin internalization on ice. (D) Hr-anxA1 internalization (green) as visualized by fluorescent confocal laser scanning microscopy (CLSM). Nuclei are stained with DAPI (blue) and PS-expression is stained with anxA5-AF568 (red). PS negative cells (indicated with white arrow) did not internalize hr-anxA1. (E) Pretreatment of PMN with 10 nM hr-anxA1 inhibited both rolling and adhesion of PMN on a TNF-α activated HUVEC monolayer. (F) One frame out of 32 is shown of a flow chamber model with rolling and adhering fluorescent THP-1 monocytes, flowing over TNF-α activated HUVEC monolayer. Rolling and adhering THP-1 monocytes are indicated by white and red arrows respectively. (G) Pretreatment of THP-1 cells with 10 nM hr-anxA1 has effect on neither rolling nor adhesion of THP-1 cells. All values are represented as mean ± SEM of 3 independent experiments.

The N-terminal tail mediates anti-inflammatory activities of anxA1 and contains protease-sensitive sites [29]. We used the expression vector pQE30Xa that introduced a His-tag upstream of the N-terminal tail, which was cleaved off after purification by FXa. In order to verify that our procedure yielded full-length recombinant product, purified hr-anxA1 was subjected to structural analyses including MALDI TOF/TOF, silver stained SDS-PAGE and Western blotting (S1 Fig) and tryptic digestion. These analyses confirmed the production and purification of full-length hr-anxA1 with a purity of >95%. Hr-anxA1 was stable for at least 1 year if stored in HEPES/NaCl pH7.4 at 4°C. Biological functionality of the N-terminal tail and Ca^2+^-dependent PS-binding of the C-terminal core were intact as assessed by the calcium flux induced in FPR-2 transfected HEK-293 cells and ellipsometry respectively (Fig 1A and 1B and S2 Fig). Furthermore, hr-anxA1 was internalized by Jurkat cells in the early phase of apoptosis in a PS-binding and FPR-dependent manner. This was demonstrated with FPR-inhibitors cyclosporine H (CsH) and WRW4 using flow cytometry (Fig 1C) and confocal microscopy (Fig 1D) and underscored by absence of internalization if hr-anxA1 lacks its N-terminus (S3 Fig). AnxA5 internalization, which depends on PS-binding and trimerization [34,35], was affected by neither inhibitor (Fig 1C). Finally, hr-anxA1 significantly inhibited rolling and adhesion of polymorphonuclear cells (50% and 63%, respectively) over a monolayer of TNFα-activated HUVECs (Fig 1E) while it had no effect on rolling and adhesion of THP-1 monocytes (Fig 1F and 1G).

### Determination of in vivo administration route of hr-anxA1

In order to establish the preferred route of administration, biodistribution and blood clearance were determined for IV and IP administered ^99m^Tc(CO)_3_-His-anxA1. ^99m^Tc(CO)_3_-His-anxA1 had radiochemical purity of approximately 90% and eluted as a single peak on RP-HPLC (S4A Fig). IV administered ^99m^Tc(CO)_3_-His-anxA1 was cleared by the kidneys, liver and lungs. IP administered ^99m^Tc(CO)_3_-His-anxA1 was gradually released into the blood circulation, peaked at 50 min post injection and then gradually decreased through clearance by the kidneys and liver (S4B–S4D Fig) with t_1/2_ of approximately 6 hours. About 0.5% of injected dose per gram (ID/g) anxA1 remained in the circulation 24 hours post injection. On basis of pharmacokinetics we decided to administer hr-anxA1 three times per week IP as treatment.

### Administration of hr-anxA1 does not alter baseline characteristics of LDLR^-/-^ mice

Treatment of LDLR^-/-^ mice on WTD with hr-anxA1 had no effect on body weight, circulating cholesterol and triglyceride levels and procoagulant activity of blood plasma (Table 1). 6 weeks of IP administration of hr-anxA1 did not cause generation of anti-hr-anxA1 antibodies (data not shown).

**Table 1:** Baseline characteristics of control and hr-anxA1 treated mice.

	Group I		Group P	
	Ctrl	hr-anxA1	Ctrl	hr-anxA1
Age (wks)	18 ±0.5	18 ±0.5	24 ±0.5	24 ±0.5
Weight (g)	26.2 ±1.9	25.8 ±1.6	29.2 ±2.4	27.9 ±1.5
Cholesterol (mM)	25.2 ±2.9	24.6 ±3.9	28.3 ±2.7	26.9 ±2.9
Triglycerides (mM)	3.3 ±0.6	3.2 ±0.8	3.3 ±0.6	3.6 ±0.5
Coagulation				
ETP (nM.min^-1^)	1376 ±104.0	1312 ±46.3	1342 ±78.1	1325 ±59.8
Peak height (nM)	185.3 ±6.7	190.8 ±11.0	180.1 ±7.3	188.6 ±8.9

### Hr-anxA1 has no significant effect on early plaque development

**Fig 2:**
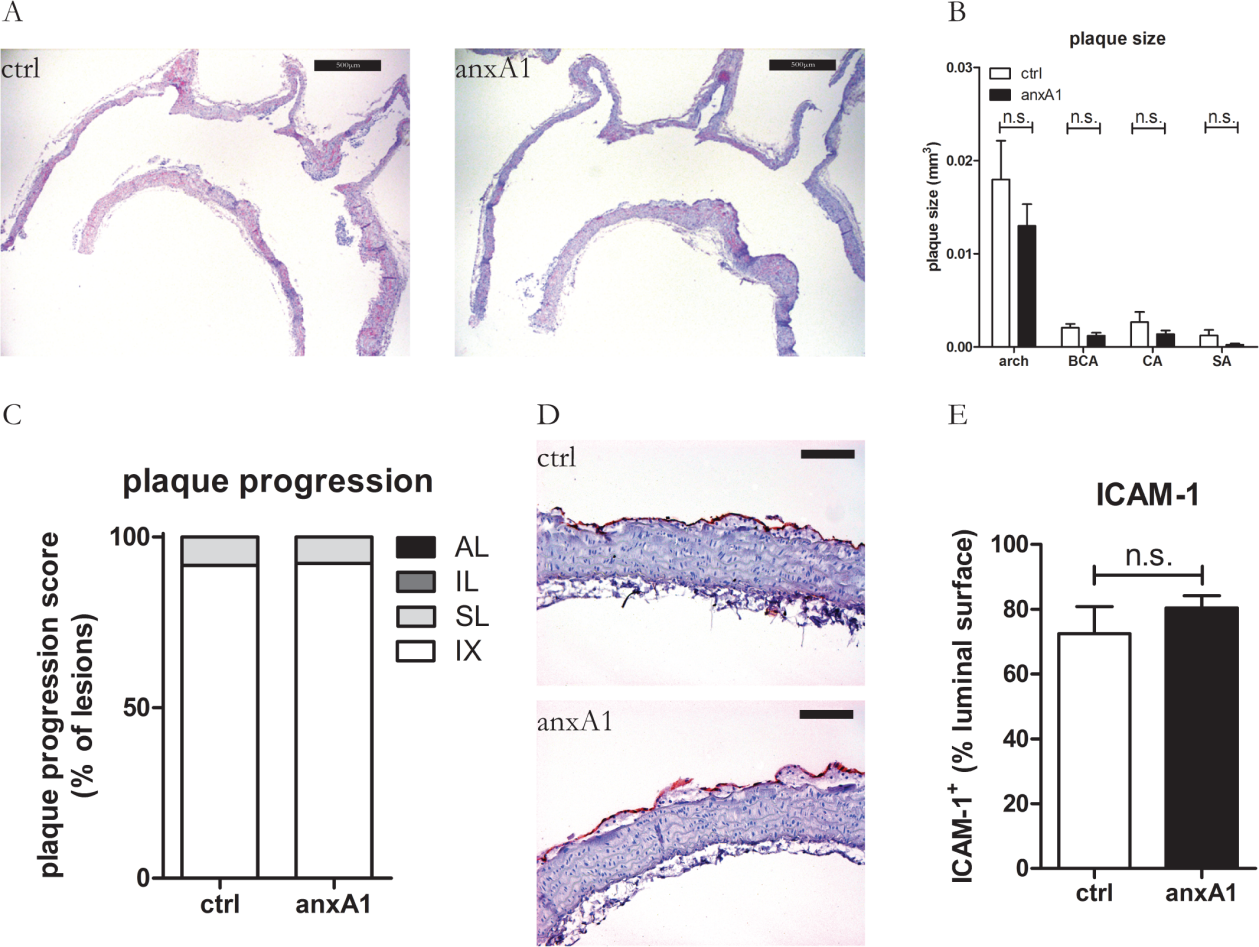
Hr-anxA1 has no significant effect on early plaque development. 12 weeks old mice were fed WTD during 6 weeks. Hr-anxA1 treatment started at start of WTD. (A) Representative H&E staining of aortic arches after 6 weeks of treatment. (B) Treatment with hr-anxA1 did not affect plaque burden in the arch and subclavian (SA) brachiocephalic (BCA) and left common carotid artery (CA). (C) Individual plaque stability and progression was scored on following parameters: neutrophil and macrophage content, apoptosis and necrosis, cap thickness and calcification status (see S1 and S2 Tables). Hr-anxA1 treatment had no effect on early plaque stability and progression. (D, E) Hr-anxA1 treatment had no effect on endothelial ICAM-1 expression of IX and SL lesions. IX: intimal xanthoma; SL: small lesion; IL: intermediate lesion; AL: advanced lesion. All values are represented as mean ±SEM, n = 12 animals per group. Panel A: 40x magnification, scale bar represents 500μm. Panel D: 200x magnification, scale bar represents 100μm.

Pharmacological treatment with hr-anxA1 starting at start of WTD (Group I) was without significant effect on plaque growth during 6 weeks of treatment (total plaque size 0.024±0.019 mm^3^ versus 0.016±0.010 mm^3^, control versus hr-anxA1, P = 0.21). Plaque growth was comparable for hr-anxA1 treated and vehicle treated mice at different aortic sites (Fig 2A and 2B). Both hr-anxA1 and vehicle treated mice developed intimal xanthoma (IX) and small foam cell lesions (SL) (Fig 2C) with infiltrated macrophages in comparable proportion, indicating that hr-anxA1 affects neither onset of IX formation nor their transition into SL in LDLR^-/-^ mice under atherogenic pressure. 70% to 80% of the endothelium of the aortic arch was activated as evidenced by ICAM-1 expression. Pharmacological treatment with hr-anxA1 had no significant effect on ICAM-1 expression (Fig 2D and 2E).

### Hr-anxA1 attenuates progression into advanced plaques

**Fig 3:**
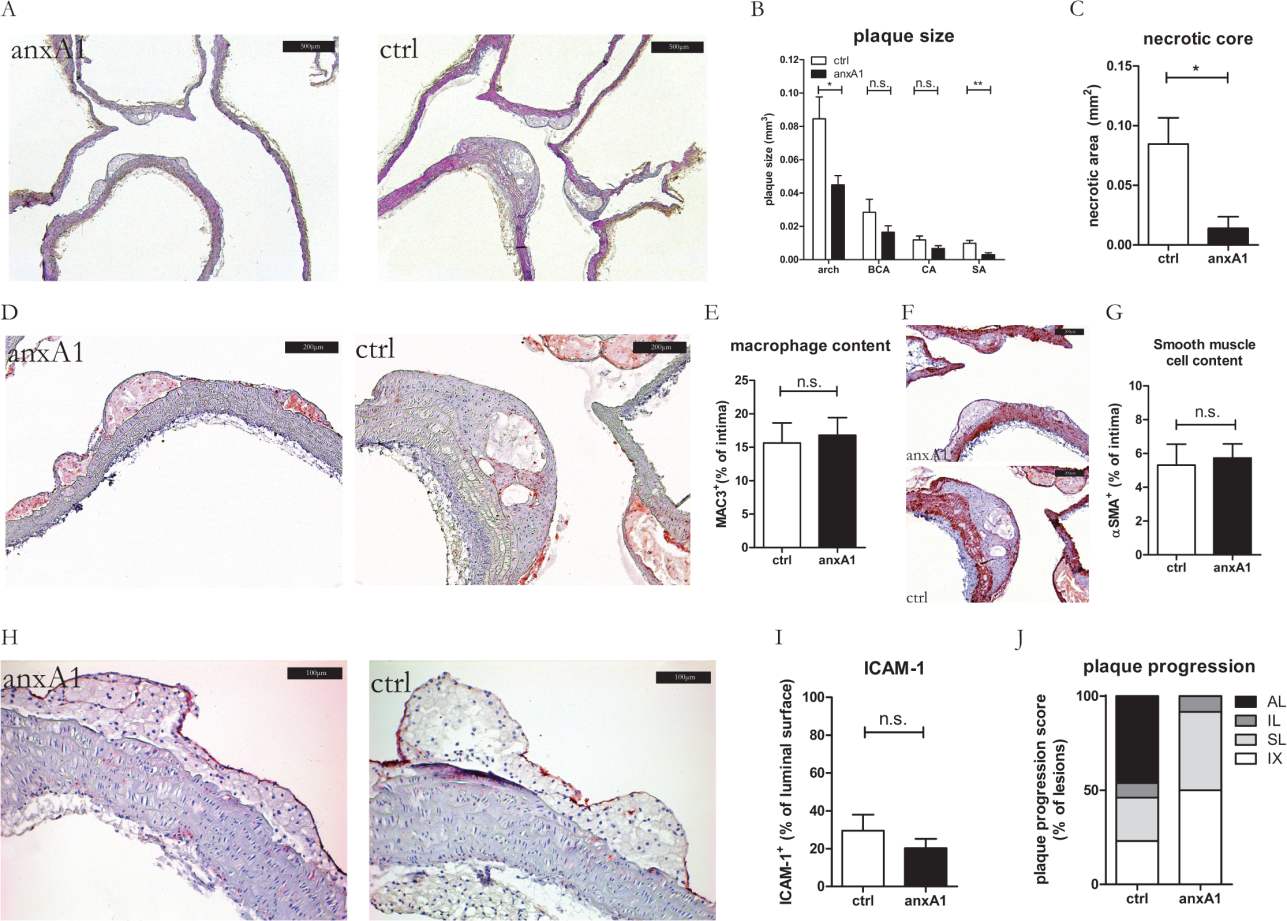
Hr-anxA1 attenuates progression into advanced plaques. (A) 12 weeks old mice were fed WTD 12 weeks. During the last 6 weeks mice were treated with hr-anxA1 or vehicle (ctrl). (A) Representative H&E staining of aortic arches. (B) Treatment with hr-anxA1 significantly reduced total plaque burden in the inner arch (arch) and subclavian artery (SA) but not in the brachiocephalic (BCA) and left common carotid artery (CA). (C) Individual plaque progression was scored on following parameters: neutrophil and macrophage content, apoptosis and necrosis, cap thickness and calcification status (see S1 and S2 Tables). (D, E) Endothelial ICAM-1 expression was reduced in early plaque development (IX/SL) after anxA1 treatment. (F, G) Macrophage and (H, I) smooth muscle cell content were comparable between anxA1 and vehicle treated controls. IX: intimal xanthoma; SL: small lesion; IL: intermediate lesion; AL: advanced lesion. All values are represented as mean ±SEM, n = 12 animals per group. Panel A: 40x magnification, scale bar represents 500μm; Panel D,F: 100x magnification, scale bar represents 200 μm; Panel H: 200x magnification, scale bar represents 100 μm.

Hr-anxA1 treatment of mice with established plaques (Group P) caused a significant inhibition of total plaque growth (0.131±0.080 mm^3^ versus 0.065±0.031 mm^3^, control versus hr-anxA1, -50%, P = 0.005), which was most pronounced in the arch and subclavian artery (Fig 3A and 3B). Individual plaque progression was further scored (see S1 and S2 Tables). This classification showed that hr-anxA1 treated mice predominantly developed early IX and SL, while the control treated mice had already IL and AL development (Fig 3C). Interestingly, aortic endothelium lost ICAM-1 expression (Fig 3D and 3E) in control mice receiving a WTD during 12 weeks as compared to control mice fed a WTD during 6 weeks (Fig 2D and 2E). Hr-anxA1 treatment further reduced endothelial ICAM-1 expression in Group P mice (Fig 3D and 3E), indicating a positive pharmacological effect of hr-anxA1 on resolution of inflammation. Hr-anxA1 treatment did not change the macrophage and smooth muscle cell content relative to lesion size as compared to the vehicle treated lesions (Fig 3F–3I). Blood analysis revealed significant increase in proportion of granulocytes of circulating leukocytes, while bone marrow showed no differences in leukocyte composition between control and hr-anxA1 treated mice (Table 2).

**Table 2:** Flow cytometry analysis of blood and bone marrow.

		Ctrl (n = 7)	hr-anxA1 (n = 6)	P
**Blood (% of viable)**				
**B-cells**		46.9 ±9.5	33.8 ±15.8	n.s
**NK-cells**		4.1 ±0.4	4.0 ±0.3	n.s.
**Granulocytes**		18.2 ±7.1	35.0 ±18.8	0.039
**Monocytes**		9.9 ±4.5	10.6 ±2.5	n.s.
	Ly6C^low^ (% of mono)	22.1 ±4.2	18.5 ±10	n.s
	Ly6C^medium^ (% of mono)	10.7 ±2.0	9.7 ±1.8	n.s
	Ly6C^high^ (% of mono)	67.2 ±5.6	71.8 ±8.3	n.s
**T-Cells**		14.2 ±1.8	12.1 ±4.9	n.s
	CD4+ (% of T-cell)	41.9 ±1.6	45.3 ±6.4	n.s
	CD8+ (% of T-cell)	58.1 ±1.6	54.7 ± 6.4	n.s
			
**Bone marrow (% of viable)**				
**B-cells**		17.8 ± 1.4	19.6 ±5.2	n.s
**NK-cells**		1.1 ±0.2	1.0 ±0.2	n.s
**Granulocytes**		36.7 ±2.0	30.2 ±9.1	n.s
**Monocytes**		14.8 ±1.5	14.3 ±2.0	n.s
	Ly6C^low^ (% of mono)	10.6 ±2.5	9.9 ±2.8	n.s
	Ly6C^medium^ (% of mono)	51.1 ±6.2	46.2 ±9.4	n.s
	Ly6C^high^ (% of mono)	38.2 ±7.8	43.8 ±10.6	n.s
**T-cells**		0.5 ±0.2	0.7 ± 0.3	n.s
**Undefined**		25.6 ±1.8	30.1 ±5.0	n.s

### Hr-anxA1 does not affect macrophage polarization in atherosclerotic plaque

**Fig 4:**
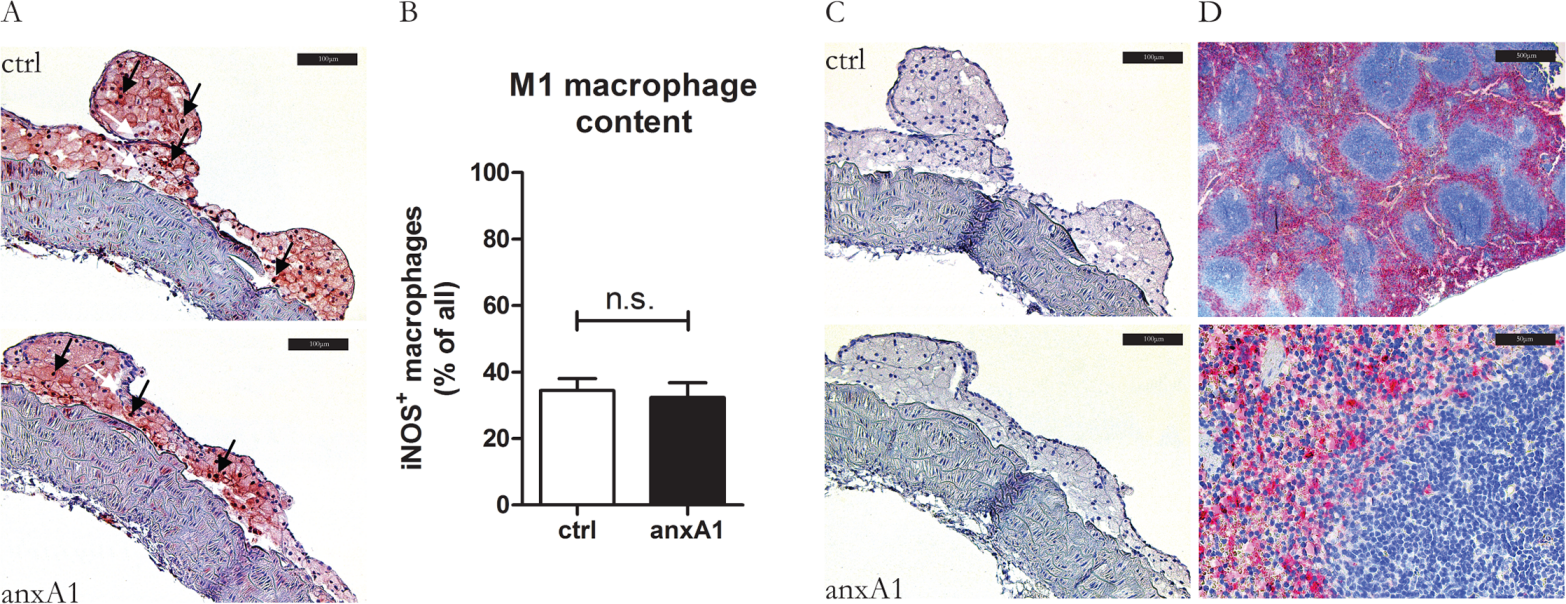
Hr-anxA1 does not affect macrophage polarization in atherosclerotic plaque. (A) Representative staining for M1 macrophage specific marker iNOS. Black arrows indicate fully commited M1 macrophages, white arrows indicate iNOS negative macrophages. (B) Quantification of iNOS-staining indicates no difference between control and anxA1 treated animals. (C) Representative staining of M2 specific marker Ym1. Both control and hr-anxA1 treated mice were negative for Ym1. (D) Staining of Ym1 positive cells in the spleen indicating functionality of anti-Ym1 antibody. All values are represented as mean ±SEM, n = 12 animals per group. Panel A: 200x magnification, scale bar represents 100μm; panel D: 40x and 400x magnification, scale bar represent 500μm and 50μm respectively.

Recently we demonstrated that endogenous anxA1 contributes to resolution of chronic inflammation in a mouse model of nonalcoholic steatohepatitis (NASH) through stimulation of IL-10 production and down-modulation of M1 polarization [36]. Therefore we studied whether hr-anxA1 could influence polarization of macrophages in developed atherosclerotic plaques (P). Quantification of M1 polarization marker iNOS (Fig 4A and 4B) showed no differences between control and anxA1 treated plaques. Staining was diffuse throughout the entire lesion, whereas the non-immune immunoglobulin staining was negative. We did not detect any marker of M2 polarization in the atherosclerotic lesions of both groups using antibodies against Ym1 (Fig 4C and 4D). This indicates that the lesions are predominantly occupied by M1 macrophages. Treatment of bone marrow-derived macrophages (BMDM) with hr-anxA1 *in vitro* did not affect IL-12, TNFα and NO release as compared to control (S6A and S6B Fig). IL-10 remained below detection limit in hr-anxA1 treated and untreated BMDM

## Discussion

AnxA1 has anti-inflammatory and pro-resolving properties causing dampening of inflammation in several animal models of acute and chronic inflammation [36–38]. Recently it was demonstrated that the anxA1-FPR2 axis suppresses atherogenesis in the apoE-/- mouse model [24]. In vivo administration of anxA1’s N-terminal peptide Ac2-26 inhibited myeloid interaction with endothelium in an FPR2-dependent manner during short-term experiments [24]. Long-term treatment with Ac2-26 peptide coupled to collagen IV-targeting nanoparticles reduced inflammation of advanced plaques of LDLR^-/-^ mice in an FPR2-dependent manner [39]. We hypothesized that full length anxA1 reduces atherogenesis if administered to LDLR^-/-^ mice on WTD. Therefore we produced recombinant human anxA1 (hr-anxA1) and thoroughly inspected integrity and functional properties of the N-terminal tail and C-terminal core. Both are essential to the anti-inflammatory properties of anxA1, since this requires both interaction with FPR2 and binding to PS, respectively [22]. Hr-anxA1 possessed both functional properties with sufficient shelf-stability to perform series of *in vivo* experiments. Secondly, pharmacokinetics and biodistribution of hr-anxA1 were determined to select the preferred route of administration. Intravenous administered hr-anxA1 was cleared rapidly from circulation with t_1/2_ of <10 min. This rapid pharmacokinetic is comparable with half-life reported for anxA5, another member of the annexin family [40]. Biodistribution showed accumulation of hr-anxA1 in kidneys but also in liver and lungs. Currently we have no explanation for the latter phenomenon but this could indicate clearance via the reticula-endothelial system. Pharmacokinetics and biodistribution of intraperitoneally administered hr-anxA1 revealed release of hr-anxA1 into circulation during a longer period of time with a peak at 50 min post administration. Here, clearance from circulation occurred predominantly by kidneys. Based on pharmacokinetics and biodistribution we decided to administer hr-anxA1 by IP injection with a dosing regime of 3 times per week 1mg/kg. The dosing regime was extrapolated from published results (38) and should be optimized by future studies.

AnxA1 suppresses and resolves inflammation predominantly through targeting neutrophils [12]. Evidence is accumulating that neutrophils play important roles in atherogenesis and contribute to initiation, progression and destabilization of atherosclerotic plaques [41–44]. Our experiments showed that treatment with hr-anxA1 was without significant effect on initiation of plaque formation but inhibited significantly plaque progression. This was most pronounced in the inner curvature of the arch and in the subclavian artery. Currently we have no explanation for the lack of a significant effect of hr-anxA1 on the early stage of atherogenesis but this could be related to the model of the LDLR^-/-^ mouse on WTD.

The N-terminal peptide of anxA1 (Ac2-26) lowers expression of ICAM-1 by atherosclerotic lesional cells [39] and reduces affinity of neutrophils and monocytes for ICAM-1 and VCAM-1 when they are activated by CCL5 [45]. ICAM-1 is a strong regulator of neutrophil adhesion and transmigration [46]. We show that full-length anxA1 reduces endothelial ICAM-1 expression in a similar manner. In addition, anxA1 can inhibit adhesion of neutrophils to endothelial cells directly [19,47] and diminish their transmigration across the endothelium [48] by engaging via its N-terminal tail with the FPR2/AXLR [14]. Concordant with a previous report [48] we observed that hr-anxA1 induced a relative increase of circulating granulocytes, which is an indication for hr-anxA1 induced neutrophil demargination and inactivation. A similar effect has been reported for treatment with glucocorticoids [49], of which anxA1 is considered to be a down-stream effector [12]. Interestingly, our *in vivo* results show that although hr-anxA1 treatment did not affect intimal xanthoma and small lesion formation it afforded substantial inhibitory effects on progression of small lesions towards advanced lesions. Recent observations showed that hyper-activation and life prolongation of neutrophils through knocking out CXCR4 affect progression rather than initiation of atherosclerosis [42]. Taken together our findings demonstrate that pharmacological treatment with full length anxA1 suppresses atherogenesis in the LDLR^-/-^ mouse model. The neutrophil has been reported as an effector of anxA1’s anti-inflammatory actions. However, hr-anxA1 may dampen the inflammatory process of atherosclerosis by acting on the plaque macrophage. We reported recently that anxA1 can polarize liver macrophages towards the anti-inflammatory M2 phenotype [50]. Macrophage polarization is an important contributor to atherogenesis [51,52]. M2 polarized macrophages diminish plaque inflammation and inhibit plaque progression [53]. However, we found no evidence of macrophage skewing in the atherosclerotic lesions of our mice. We cannot exclude that hr-anxA1 modulates macrophage function in other ways. For example hr-anxA1 may enhance efferocytosis by macrophages. Apoptosis and efferocytosis determine rate of plaque progression and plaque phenotype [54,55]. Hr-anxA1 can act as a bridging molecule between the apoptotic cell and the macrophage and, thereby, it stimulates efferocytosis [22,56,57] and reduces release of the pro-inflammatory IL-6 and TNF-α [58]. In our experiments we observed that atherosclerotic plaques treated with hr-anxA1 had smaller necrotic cores, suggestive of enhanced efferocytosis.

We conclude that progression of atherosclerosis can be attenuated pharmacologically with hr-anxA1 in a murine model of atherosclerosis. These findings may open novel avenues to treat the chronic inflammatory disease atherosclerosis.

## Supporting Information

S1 Fig Production and purification of hr-anxA1. (A) MALDI-TOF/TOF analysis shows a molecular weight of 38.5kDa for purified hr-anxA1, which is in concordance with the theoretical molecular weight of full-length anxA1. Bis-protonated hr-anxA1 and hr-anxA1 dimers are represented on the spectrum as peaks of 19.2kDa and 77.2kDa respectively. (B) Representative image of 50ng purified hr-anxA1 (1) and His-anxA1 (2) on silver-stained SDS-PAGE for total protein and (C) Western blotting with an α-anxA1-antibody (n ≥ 5 purifications) M = broad range protein marker.
(TIF)

S2 Fig Ellipsometry analysis of phospholipid binding properties. (A) Ca^2+^-dependent binding curves of 1 µg/ml hr-anxA1 and 1 µg/ml anxA5 binding to 5/95, (B) 10/90 and (C) 20/80 mol% PS/PC bilayer as measured by ellipsometry.
(TIF)

S3 Fig MALDI-TOF/TOF analysis of truncated hr-anxA1. Mass spectrometry analysis of truncated hr-anxA1 shows a molecular weight of 35.8kDa, which means truncation occurred between Thr^23^ en Val^24^.
(TIF)

S4 Fig Determination of in vivo administration route of hr-anxA1. (A) HPLC analysis of radio-labeled His-anxA1 (^99m^Tc(CO)_3_-His-anxA1) shows a radiochemical purity of >95% and specific retention time of approximately 18 minutes. (B) SPECT image 45 minutes after intraperitoneal (IP) and intravenous (IV) injection of ^99m^Tc(CO)_3_-His-anxA1. White arrows indicate site of injection, red arrows indicate kidneys and green arrows indicate lungs. Rapid renal clearance and uptake in the lungs was observed in the IV injected mice, whereas IP injection mice show peritoneal localization. (C) Time courses of blood levels of ^99m^Tc(CO)_3_-His-anxA1 were determined by γ-counting. (D) Biodistribution was determined by weighing and γ-counting of organs dissected 3 and (E) 24 hours post-injection. All values are represented as mean ± SEM, n = 6 animals per group.
(TIF)

S5 Fig Correlation of 2 and 3 dimensional plaque sizes. Plaque volumes were determined and correlated with plaque areas of the section having the largest plaque area.
(TIF)

S6 Fig Bone marrow derived macrophage response to anxA1. Bone marrow derived monocytes were differentiated to macrophages and stimulated with 1 or 10 μg/ml anxA1. (A) Release of IL-12 and TNF-α and (B) nitric oxide were measured and showed no differences between control and anxA1 addition. All values are represented as mean ± SEM (n = 4 animals).
(TIF)

S1 Table Quantification of plaque progression. (DOCX)

S2 Table Definition of plaque progression score. (DOCX)
