# Supplementary figures and images for: Pharmacological Treatment with Annexin A1 Reduces Atherosclerotic Plaque Burden in LDLR-/- Mice on Western Type Diet
Source: PLoS One. 2015 Jun 19;10(6):e0130484. doi: 10.1371/journal.pone.0130484 (PMC4475013; doi:10.1371/journal.pone.0130484)

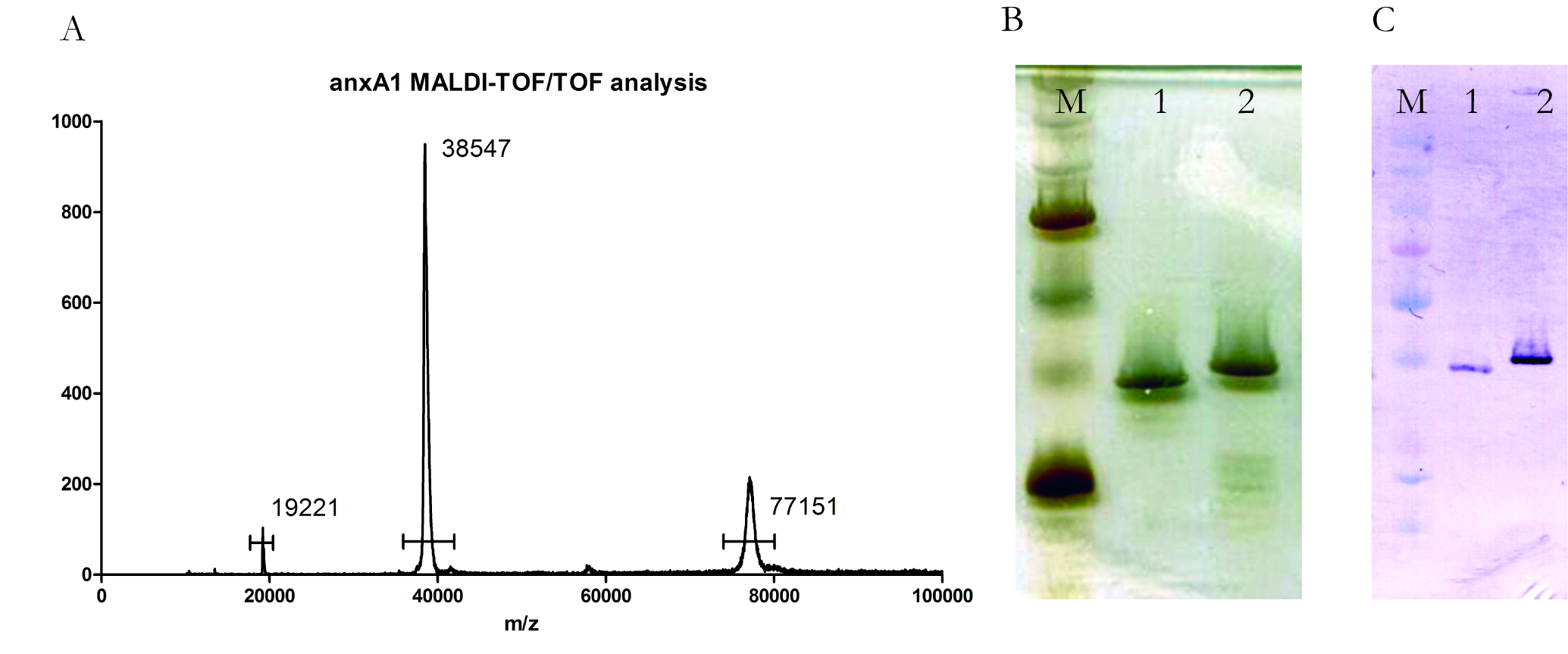

Supplement: S1 Fig — (A) MALDI-TOF/TOF analysis shows a molecular weight of 38.5kDa for purified hr-anxA1, which is in concordance with the theoretical molecular weight of full-length anxA1. Bis-protonated hr-anxA1 and hr-anxA1 dimers are represented on the spectrum as peaks of 19.2kDa and 77.2kDa respectively. (B) Representative image of 50ng purified hr-anxA1 (1) and His-anxA1 (2) on silver-stained SDS-PAGE for total protein and (C) Western blotting with an α-anxA1-antibody (n ≥ 5 purifications) M = broad range protein marker. (TIF) [file pone.0130484.s001.tif]

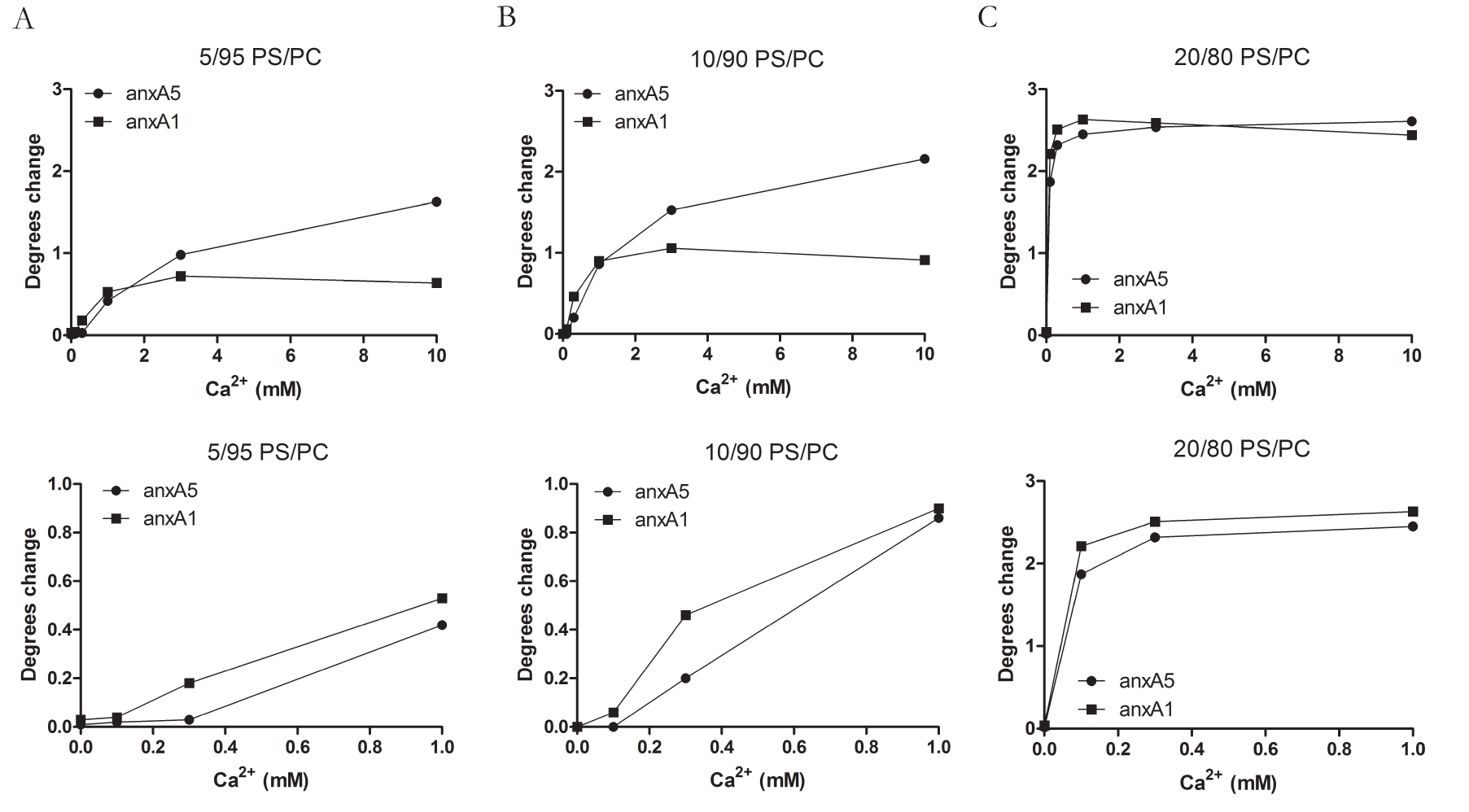

Supplement: S2 Fig — (A) Ca2+-dependent binding curves of 1 µg/ml hr-anxA1 and 1 µg/ml anxA5 binding to 5/95, (B) 10/90 and (C) 20/80 mol% PS/PC bilayer as measured by ellipsometry. (TIF) [file pone.0130484.s002.tif]

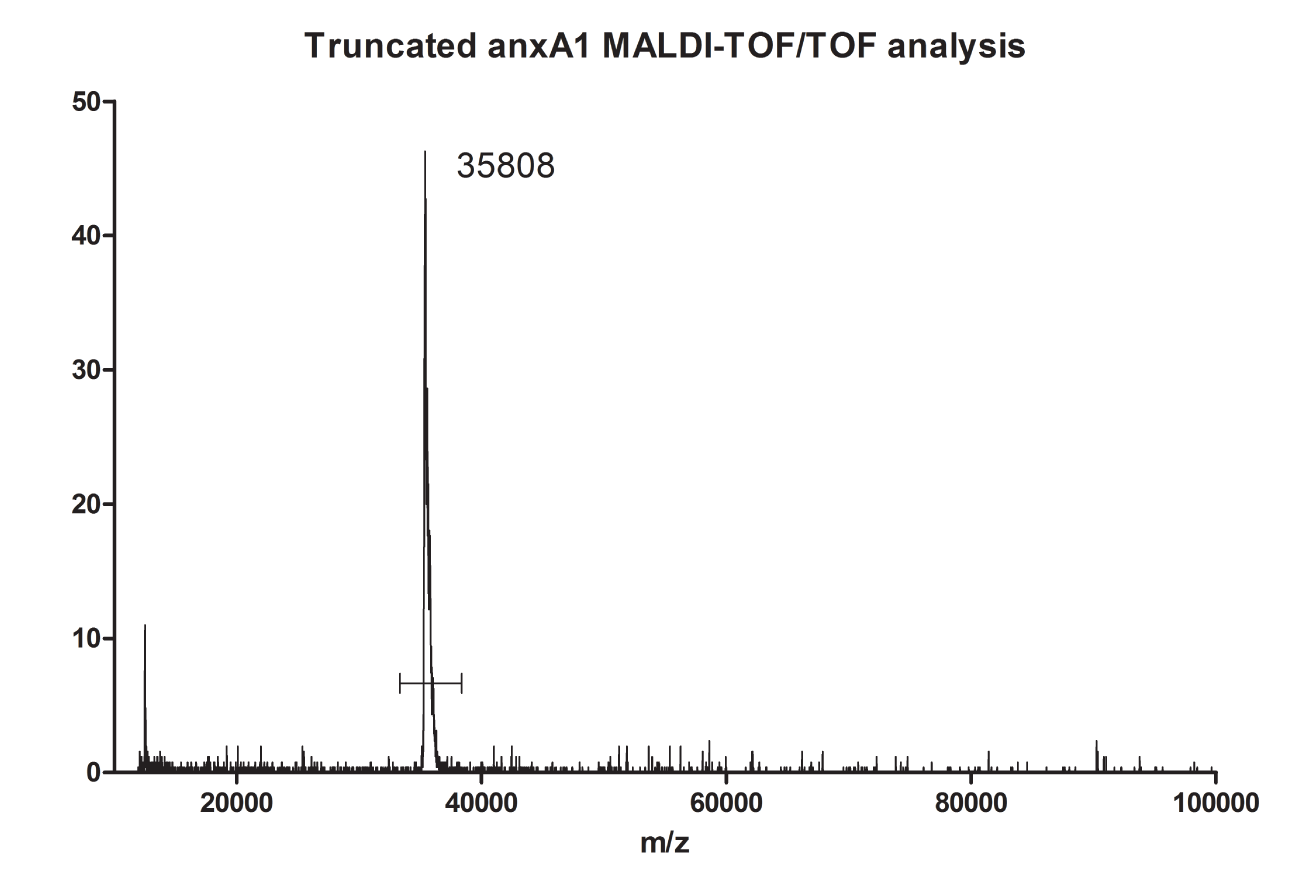

Supplement: S3 Fig — Mass spectrometry analysis of truncated hr-anxA1 shows a molecular weight of 35.8kDa, which means truncation occurred between Thr23 en Val24. (TIF) [file pone.0130484.s003.tif]

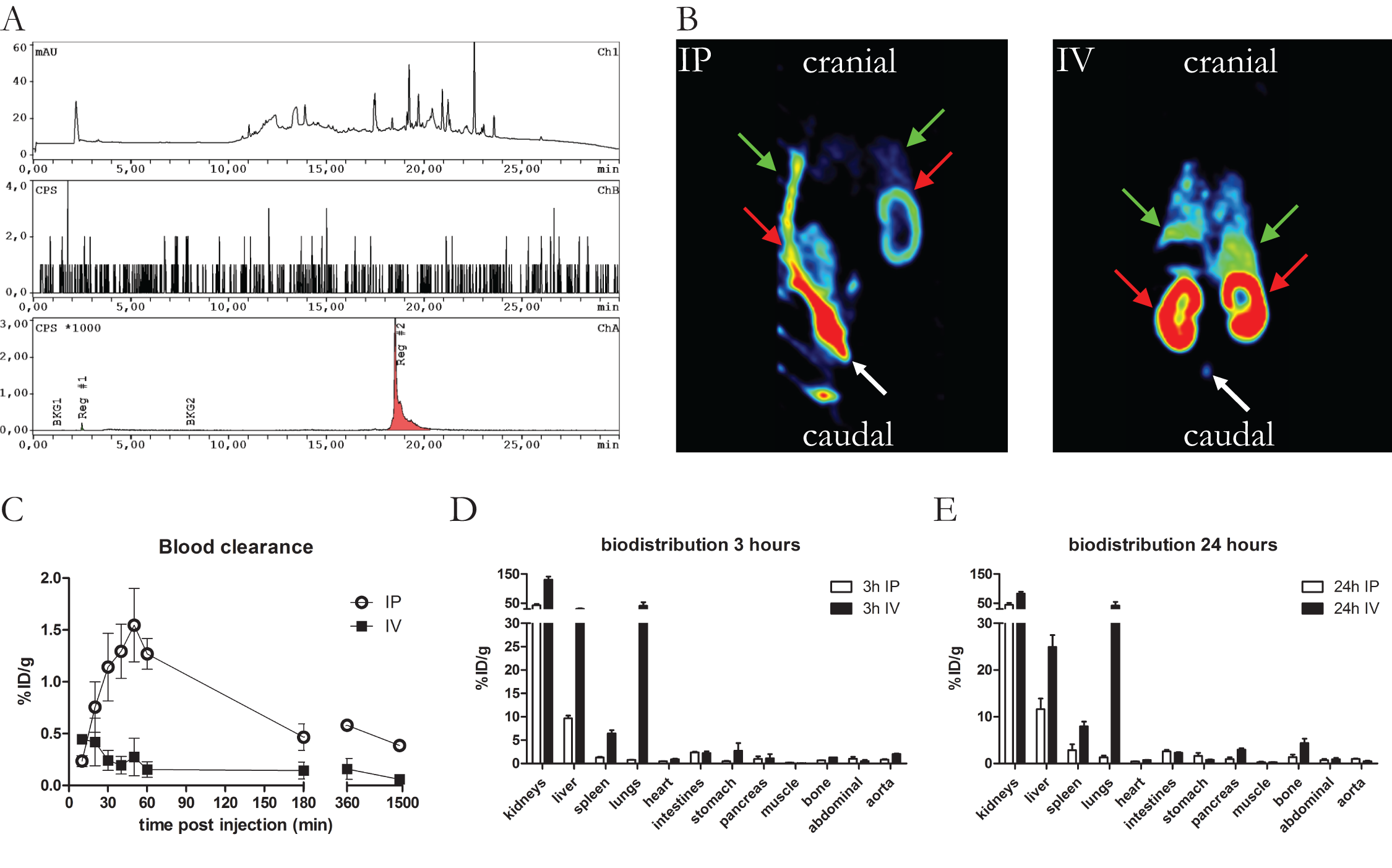

Supplement: S4 Fig — (A) HPLC analysis of radio-labeled His-anxA1 (99mTc(CO)3-His-anxA1) shows a radiochemical purity of >95% and specific retention time of approximately 18 minutes. (B) SPECT image 45 minutes after intraperitoneal (IP) and intravenous (IV) injection of 99mTc(CO)3-His-anxA1. White arrows indicate site of injection, red arrows indicate kidneys and green arrows indicate lungs. Rapid renal clearance and uptake in the lungs was observed in the IV injected mice, whereas IP injection mice show peritoneal localization. (C) Time courses of blood levels of 99mTc(CO)3-His-anxA1 were determined by γ-counting. (D) Biodistribution was determined by weighing and γ-counting of organs dissected 3 and (E) 24 hours post-injection. All values are represented as mean ± SEM, n = 6 animals per group. (TIF) [file pone.0130484.s004.tif]

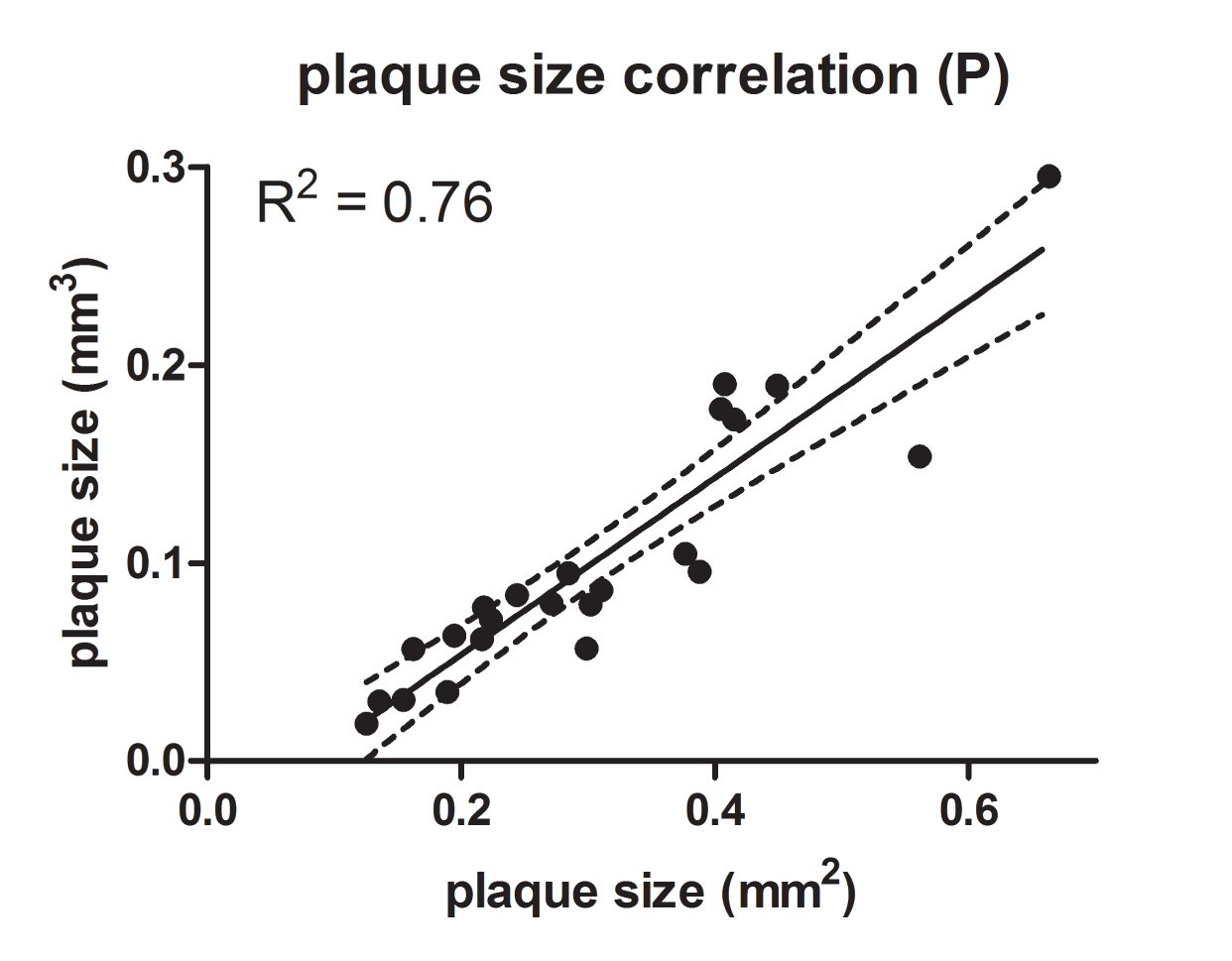

Supplement: S5 Fig — Plaque volumes were determined and correlated with plaque areas of the section having the largest plaque area. (TIF) [file pone.0130484.s005.tif]

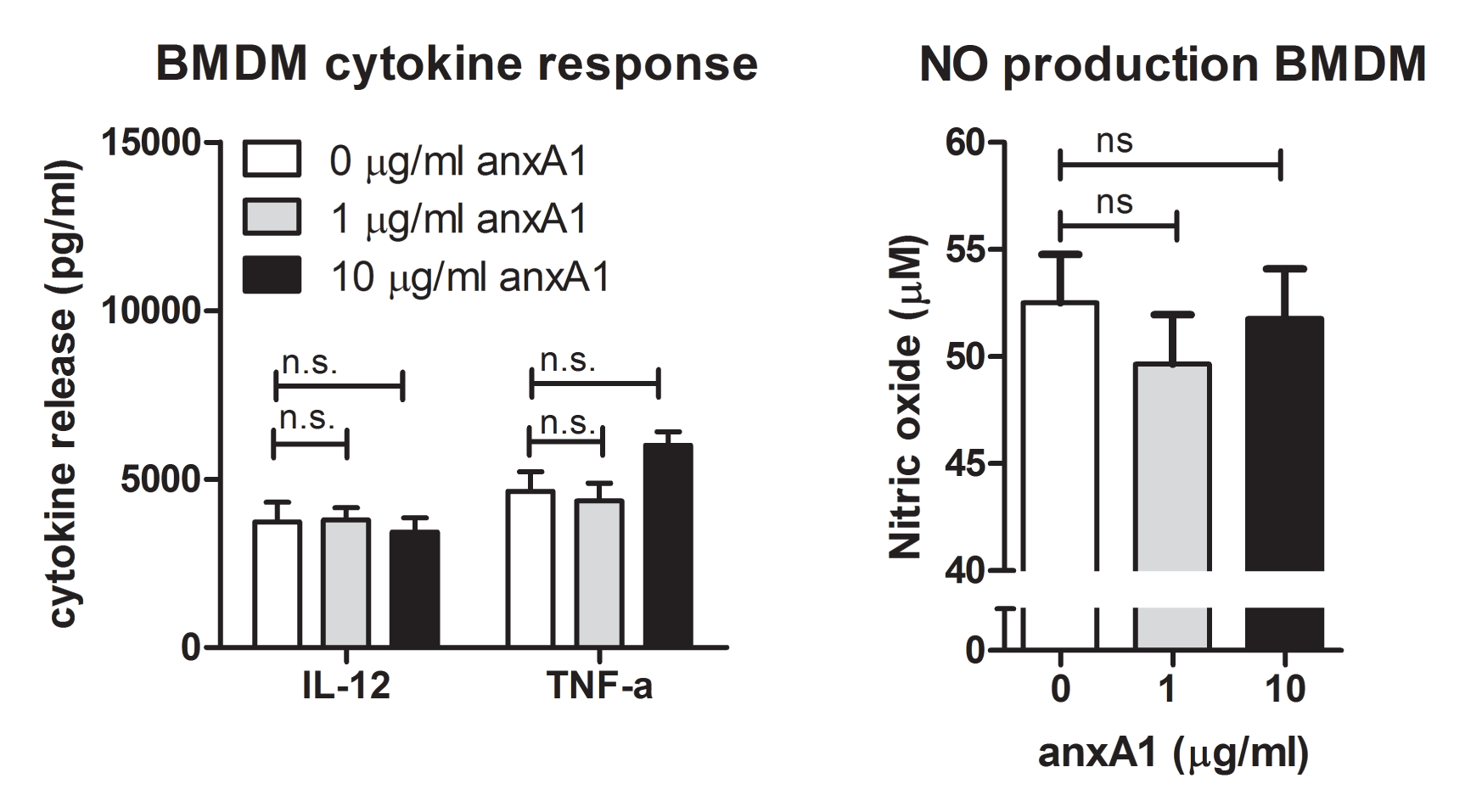

Supplement: S6 Fig — Bone marrow derived monocytes were differentiated to macrophages and stimulated with 1 or 10 μg/ml anxA1. (A) Release of IL-12 and TNF-α and (B) nitric oxide were measured and showed no differences between control and anxA1 addition. All values are represented as mean ± SEM (n = 4 animals). (TIF) [file pone.0130484.s006.tif]
