# Supplementary material for: Pharmacological Treatment with Annexin A1 Reduces Atherosclerotic Plaque Burden in LDLR-/- Mice on Western Type Diet
Source: PLoS One. 2015 Jun 19;10(6):e0130484. doi: 10.1371/journal.pone.0130484 (PMC4475013; doi:10.1371/journal.pone.0130484)
Supplement: S1 Table — (DOCX) [file pone.0130484.s007.docx]

**Supplemental Table 1 – quantification of plaque progression**

| **subject** | **amount** | **score** |
| --- | --- | --- |
| Neutrophils (#) | 0 | 0 |
|  | 1,2 | 1 |
|  | ≥3 | 2 |
| Macrophages (% intima) | 0-10 | 0 |
|  | 11-20 | 1 |
|  | 21-30 | 2 |
|  | ≥31 | 3 |
| Apoptosis (yes/no) | negative | 0 |
|  | positive | 1 |
| Necrotic core size (% intima) | 0 | 0 |
|  | <20% | 1 |
|  | ≥20% | 2 |
| Cap thickness (cell layers) | 0,1 | 0 |
|  | 2,3 | 1 |
|  | >3 | 2 |
| Calcification (status) | absent | 0 |
|  | osteochondrocytic cells | 1 |
|  | calcification | 2 |
