# Supplementary material for: Pharmacological Treatment with Annexin A1 Reduces Atherosclerotic Plaque Burden in LDLR-/- Mice on Western Type Diet
Source: PLoS One. 2015 Jun 19;10(6):e0130484. doi: 10.1371/journal.pone.0130484 (PMC4475013; doi:10.1371/journal.pone.0130484)
Supplement: S2 Table — (DOCX) [file pone.0130484.s008.docx]

**Supplemental Table 2 – Definition of plaque progression score**

| **Total score** | **definition** | **abbreviation** |
| --- | --- | --- |
| 0-3 | Intimal xanthoma | IX |
| 4-5 | Small lesion | SL |
| 6-7 | Intermediate lesion | IL |
| ≥8 | Advanced lesion | AL |
